# Impact of inorganic nitrate on renal sodium excretion during saline loading in healthy adults; secondary endpoints from a randomized crossover trial

**DOI:** 10.14814/phy2.70919

**Published:** 2026-06-04

**Authors:** A. M. Østergaard, J. B. Rosenbæk, J. A. Ejlersen, F. H. Mose, J. N. Bech, M. H. Vrist

**Affiliations:** ^1^ University Clinic in Nephrology and Hypertension Gødstrup Hospital Herning Denmark; ^2^ Department of Nuclear Medicine Gødstrup Hospital Herning Denmark; ^3^ Department of Clinical Medicine Aarhus University Aarhus C Denmark

**Keywords:** arginine vasopressin, hemodynamic, inorganic nitrate, nitric oxide, renal sodium excretion, saline loading

## Abstract

Inorganic nitrate may lower blood pressure (BP) and enhance natriuresis through nitric oxide (NO) mediated mechanisms. Studies have found inconsistent effects of NO on sodium excretion. Nitric oxide synthase (NOS) inhibition suggests increased NO promotes natriuresis, whereas inorganic nitrate studies remain inconclusive. We conducted a randomized, double‐blind, placebo‐controlled crossover trial in 18 healthy adults, with 4 days of potassium nitrate (KNO_3_) or placebo before 1 L isotonic saline infusion. BP, hemodynamic markers of the NO system, and renal function were measured. After saline GFR, renal water and sodium handling did not significantly differ between KNO_3_ and placebo (KCl). Central diastolic BP was modestly higher during KNO_3_ (*p* = 0.023). Brachial systolic BP did not differ (*p* = 0.052); however, systolic BP was higher during KNO_3_ after 120 min (Sidak adjusted, *p* = 0.030). Plasma levels of the second messenger of NO, cyclic guanosine monophosphate (cGMP), were lower after saline during KNO_3_ (*p* = 0.012). Short‐term KNO_3_ did not induce a natriuretic response during acute saline loading. KNO_3_ was associated with a small increase in central diastolic BP and lower plasma cGMP levels. These findings suggest that high‐dose KNO_3_ exposure may affect the endogenous NO response.

## 1 INTRODUCTION

Inorganic nitrate has been shown to lower blood pressure (BP) in both healthy individuals and patients with hypertension (Kapil et al., 2010; Kapil et al., 2015). This effect is thought to be mediated by nitric oxide (NO), through the sequential reduction of nitrate (NO_3_^−^) to nitrite (NO_2_^−^), and subsequently to NO. Endogenous nitric oxide production is promoted by endothelial shear stress via nitric oxide synthase (NOS)‐mediated conversion of L‐arginine (Potter, 2011). The resulting NO formation primarily induces vasodilation but has also been shown to elicit important renal effects through natriuresis and diuresis (Gabbai & Blantz, 1999). However, studies on NO and natriuresis have reported variable results. Investigations involving NOS inhibition have demonstrated decreased sodium excretion suggesting a natriuretic effect of endogenous nitric oxide (Bech et al., 1996; Kielstein et al., 2004; Larsen et al., 2012). In contrast, studies involving various NO donors, such as sodium nitroprusside and L‐arginine, have produced more inconsistent findings (Kanno et al., 1992; Nielsen et al., 1993). Thus, it remains uncertain whether the administration of an NO donor increases sodium excretion. An acute volume load induces endothelial shear stress. Increased shear stress stimulates endothelial NO release, which in turn promotes arterial relaxation and thereby prevents a sudden rise in BP (Sriram et al., 2016). Through these actions, NO contributes to the reduction of endothelial shear stress and buffers the hemodynamic effects of acute volume loading (Gabbai & Blantz, 1999; Sriram et al., 2016). Furthermore, an acute volume expansion is known to stimulate low‐pressure baroreceptors and cardiopulmonary mechanoreceptors, which can regulate sympathetic tone (Charkoudian et al., 2004; Convertino et al., 1984; Mancia & Grassi, 2014). Arginine vasopressin (AVP) secretion is also regulated by both baroreceptor activity, with e.g. atrial stretch suppressing its release. Consequently, an acute volume load would reduce AVP secretion and thereby decrease renal water reabsorption, promoting diuresis and a reduction in plasma volume, contributing to the maintenance of volume homeostasis during an acute volume expansion (Charkoudian et al., 2004; Convertino et al., 1984; Mancia & Grassi, 2014). Experimental studies have previously shown that NO donors and NOS inhibition can modulate AVP release (Eriksson et al., 1982; Goyer et al., 1994; Lutz‐Bucher & Koch, 1994; Manning Jr. et al., 1994; Ota et al., 1993; Yasin et al., 1993). We have previously shown, in this randomized crossover study of healthy participants, that 4 days of potassium nitrate (KNO_3_) did not alter BP or renal water and sodium excretion compared with placebo (Østergaard et al., 2023).

To assess whether more subtle or compensatory changes might become evident during acute saline loading, we examined the responses to an isotonic saline infusion. We hypothesized that KNO_3_ treatment would increase NO bioavailability and enhance natriuresis while attenuating the BP response during saline loading. Contrary to our hypothesis, KNO_3_ did not enhance renal or hemodynamic responses to saline loading but was associated with modest changes in the opposite direction.

## 2 MATERIALS AND METHODS

We have previously published a demographic overview and a detailed description of the study design (Østergaard et al., 2023). This report represents the secondary endpoints of a randomized, double‐blind, placebo‐controlled crossover trial including 18 healthy adults (NCT04755400).

### 2.1 Ethics

This study was approved by the Regional Committee on Biomedical Research Ethics (case number: 1‐10‐72‐96‐16). Informed, signed consent was obtained from each patient. The study was carried out in accordance with the Declaration of Helsinki.

### 2.2 Design

In a randomized crossover design, participants completed two 4‐day treatment periods with either KNO_3_ capsules (24 mmol/day, 1488 mg) or placebo capsules (24 mmol/day potassium chloride, KCl) separated by a washout period of at least 4 weeks. At the end of each treatment period, participants underwent an examination day with an intravenous saline infusion.

### 2.3 Endpoints

Assay methods and device specifications have been published previously (Østergaard et al., 2021, 2023).

#### 2.3.1 Renal endpoints

Glomerular filtration rate (GFR), urinary sodium excretion, fractional sodium excretion, fractional potassium excretion, free water clearance, urinary epithelial sodium channel γ‐subunit (u‐ENaCγ), and urinary Na–Cl cotransporter (u‐NCC).

#### 2.3.2 Vasoactive hormones

Plasma renin concentration, angiotensin II, aldosterone, AVP, and brain natriuretic peptide (BNP).

#### 2.3.3 NO pathway

Plasma nitrate, urinary nitrate, plasma nitrite, urinary nitrite, and plasma cyclic guanosine monophosphate (cGMP).

#### 2.3.4 Systemic hemodynamic

Heart rate, brachial BP, and central BP.

### 2.4 Study medication

Capsules were manufactured with similar appearances containing 24 mmol (1488 mg) KNO_3_ or KCl (Glostrup Pharmacy, Glostrup, Denmark). Blinding, packing, and randomization of the capsules was performed by the hospital pharmacy, as previously described (Østergaard et al., 2023). Isotonic saline (0.9%) was manufactured by B. Braun Medical A/S (Melsungen, Germany).

### 2.5 Experimental procedure

#### 2.5.1 Prior to examination day

In 4 days prior to each examination day, the subjects had a standardized diet, and they were treated with either KNO_3_ or KCl capsules (Østergaard et al., 2023).

#### 2.5.2 Examination day with intravenous saline infusion

On the examination day, the final capsule dose was administered at 06:00 am. Two peripheral venous catheters were inserted. One dedicated to tracer infusion and the other for blood sampling and saline administration. Baseline values (time 0) were defined as the mean of pre‐infusion measurements obtained between 08:30 and 11:30 am. Subsequently, 1 L of isotonic saline was infused intravenously over a 20‐min period (11:30–11:50 am; time 0–20 min). Throughout the examination, participants remained in a supine position in a temperature‐controlled room (21°C–25°C). Urine voiding was performed while either seated or standing. To maintain hydration, participants received 175 mL of tap water every 30 min starting at 07:45 am.

Non‐invasive hemodynamic measurements using the Mobil‐O‐Graph® device were obtained and blood and urine samples were collected every 30 min from 11:30 to 13: 30 am.

### 2.6 Biochemical analyses

Methods have previously been described and are briefly summarized here (Østergaard et al., 2023). Glomerular filtration rate (GFR) was determined by the constant infusion technique using ^51^Cr‐EDTA in the initial three subjects and subsequently ^99m^Tc‐DTPA as a validated alternative (Østergaard et al., 2021).

Routine biochemical analyses, including plasma potassium, sodium, albumin, creatinine, and BNP, were performed at the Department of Clinical Biochemistry, Gødstrup Hospital, Denmark.

Plasma and urine osmolality were determined by freeze‐point depression (Advanced Instruments, Norwood, MA, USA). Angiotensin II (angII), vasopressin (AVP), Plasma renin concentration (PRC), aldosterone, urinary ENaCγ, and NCC were determined by radioimmunoassay (Al Therwani et al., 2014; Frank et al., 2020; Jensen et al., 2013; Jensen et al., 2014; Mose et al., 2015; Mose et al., 2019; Pedersen et al., 1984; Pedersen et al., 2001). Plasma and urinary cGMP were determined using a competitive enzyme immunoassay (R&D Systems, Minneapolis, MN, USA). Plasma and urinary nitrate/nitrite were determined using a nitric oxide analyzer (NOA 280i, Zysense, Frederick, CO, USA) based on ozone chemiluminescence. All samples were processed and stored under standardized conditions prior to analysis. Free water clearance (CH_2_O) and fractional excretions of sodium and potassium were calculated using standard formulas.

### 2.7 Statistical analysis

Repeated‐measures outcomes were analyzed using linear mixed‐effects models with fixed effects for treatment (KNO_3_ vs. placebo), time (baseline, 30, 60, 90, and 120 min), sex, treatment period, and the treatment × time interaction. A subject‐specific random intercept was included to account for repeated measurements within individuals. Models were fitted by restricted maximum likelihood. Inference on fixed effects was based on the Kenward‐Roger approximation. Estimated marginal means (EMMs) with 95% confidence intervals were used for all reported values and graphical presentations. Pre‐specified pairwise comparisons between treatments at individual time points were performed using contrasts of EMMs with Sidak adjustment to explore time‐specific differences. For hemodynamic and renal outcomes, contrasts were based on model‐estimated absolute values at each time point. For vasoactive hormones, additional analyses were performed using changes from baseline to evaluate treatment‐related differences in hormonal responses to saline loading. All time‐specific comparisons were interpreted cautiously in the context of the overall mixed‐model results. Missing data were assumed missing at random and handled within the mixed‐model framework. A two‐sided *p*‐value <0.05 was considered statistically significant. Analyses were performed in R (version 4.5.0) using the tidyverse, lme4, lmerTest, emmeans, ggplot2, and patchwork packages.

## 3 RESULTS

Marked differences in plasma nitrate and plasma nitrite concentrations were observed following the treatment period with KNO_3_ compared with placebo. Plasma nitrate concentrations were several folds higher after KNO_3_ treatment than after placebo (KNO_3_: ~580 μmol/L; placebo: ~10 μmol/L), and plasma nitrite concentrations were more than threefold higher (KNO_3_: ~0.37 μmol/L; placebo: ~0.14 μmol/L). These differences persisted throughout the post‐saline infusion period (*p* < 0.001).

### 3.1 Demographics

Of 28 screened subjects, 22 were included and 18 completed the study. Four were excluded from GFR assessment due to incomplete voiding, leaving 14 for analysis. Baseline characteristics are shown in Table 1.

**TABLE 1:** Baseline characteristics of participants (*n* = 18).

Baseline characteristics	
Male, % (*n*)	50 (9)
Age (years)	29 [25; 56]
Body mass index (BMI, kg/m^2^)	24.1 ± 1.9
Systolic BP (mmHg)	125 ± 7
Diastolic BP (mmHg)	76 ± 6
Heart rate (bpm)	65 ± 9
p‐creatinine (μmol/L)	75 ± 14
eGFR (mL/min/1.73 m^2^)	102 ± 16

*Note*: Values are presented as mean ± SD or medians [IQR].

### 3.2 Glomerular filtration rate and tubular function

**FIGURE 1:**
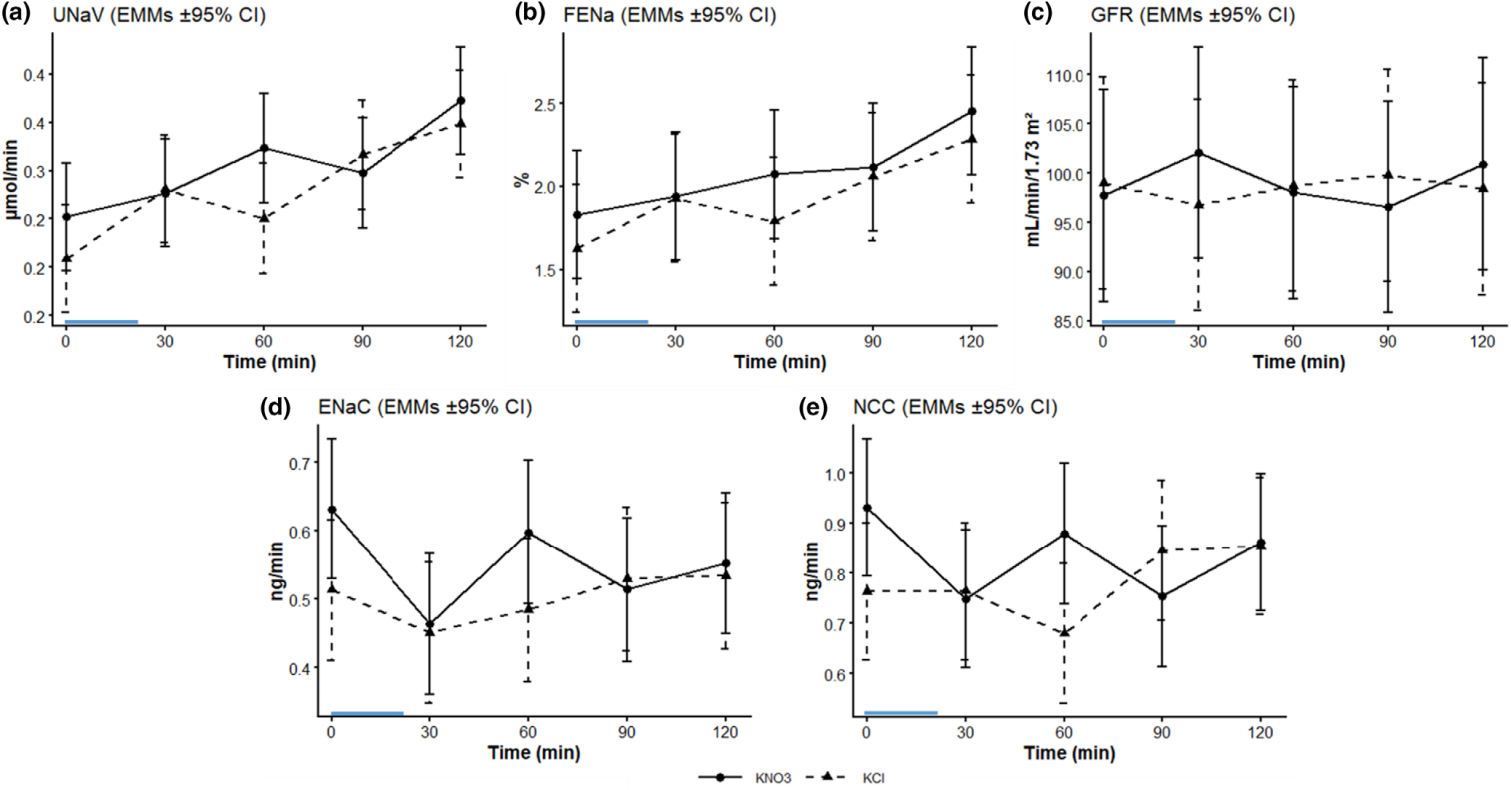
Glomerular filtration rate and renal excretory responses to acute saline infusion following 4 days of potassium nitrate versus placebo. The 1 L isotonic saline infusion was administered from 0 to 20 min. Time courses are presented as estimated marginal means ± 95% confidence intervals for (a) urinary sodium excretion (UNaV), (b) fractional sodium excretion (FE_Na_), (c) glomerular filtration rate (GFR), (d) urinary ENaCγ excretion, and (e) urinary NCC excretion, measured at baseline and at 30, 60, 90, and 120 min (*n* = 14). The shaded bar indicates the saline infusion period. Solid lines represent potassium nitrate and dashed lines represent placebo; vertical bars indicate 95% confidence intervals. Estimated marginal means were derived from linear mixed‐effects models including treatment, time, treatment period, sex, and the treatment × time interaction, with a subject‐specific random intercept. No post hoc comparisons are displayed in the figures.

Figure 1, Table 2 illustrate renal functional responses to saline infusion. Glomerular filtration rate remained stable after the infusion. Urinary sodium excretion and fractional sodium excretion were not significantly different between KNO_3_ and placebo treatment post‐infusion. After saline, fractional potassium excretion showed a non‐significant tendency toward higher values during KNO_3_ treatment (*p* = 0.058). Urinary ENaCγ excretion was not significantly different between treatments post‐infusion (*p* = 0.065), and no differences were observed for urinary NCC excretion (*p* = 0.155). Urine output and free water clearance did not differ between KNO_3_ and placebo after saline (*p* = 0.552 and *p* = 0.936, respectively).

**TABLE 2:** Effects of potassium nitrate versus placebo during saline loading on kidney function, tubular transport, systemic hemodynamics, and vasoactive hormones.

	Potassium nitrate	Placebo	Mean difference (95% CI)	Treatment	Time	Treatment × time
GFR and tubular function during saline loading (*n* = 14)						
GFR (mL/min/1.73 m2)	99 (89, 109)	99 (89, 108)	0.52 (−3.3, 4.4)	0.788	0.979	0.621
Urine output (mL/min)	7.6 (7.0, 8.3)	7.4 (6.8, 8.1)	0.2 (−0.5, 1.0)	0.552	0.223	0.366
CH_2_O (mL/min)	3.6 (3.2, 4.0)	3.6 (3.2, 4.0)	0.02 (−0.50, 0.60)	0.936	0.103	0.753
UNaV (μmol/min)	0.30 (0.27, 0.35)	0.28 (0.24, 0.32)	0.020 (−0.004, 0.050)	0.093	**<0.001**	0.244
UKV (μmol/min)	0.11 (0.10, 0.13)	0.11 (0.10, 0.12)	0.006 (−0.004, 0.015)	0.250	**<0.001**	0.063
FENa (%)	2.1 (1.8, 2.4)	1.9 (1.6, 2.3)	0.15 (−0.03, 0.32)	0.095	**<0.001**	0.839
FE_K_ (%)	26 (22, 30)	23 (19, 27)	2.1 (−0.1, 4.3)	0.058	**<0.001**	0.826
u‐ENaC (ng/min)	0.55 (0.48, 0.63)	0.50 (0.43, 0.58)	0.050 (−0.003, 0.103)	0.065	0.071	0.366
u‐NCC (ng/min)	0.84 (0.74, 0.93)	0.78 (0.69, 0.88)	0.054 (−0.021, 0.129)	0.155	0.360	0.071
Systemic hemodynamic during saline loading (*n* = 18)						
Brachial SBP (mmHg)	118 (114, 121)	116 (112, 120)	1.5 (−0.01, 3.0)	0.052	0.332	0.190
Brachial DBP (mmHg)	72 (69, 76)	72 (68, 75)	0.4 (−0.6, 1.4)	0.436	0.148	0.311
Central SBP (mmHg)	109 (105, 112)	108 (104, 111)	0.8 (−0.8, 2.3)	0.335	0.747	0.595
Central DBP (mmHg)	74 (70, 77)	72 (69, 76)	1.2 (0.2, 2.3)	**0.023**	0.193	0.540
Heart rate (beats pr. min)	59 (55, 63)	59 (55, 63)	−0.04 (−0.99, 0.91)	0.932	**0.001**	0.802
Vasoactive hormones during saline loading (*n* = 18)						
p‐aldosterone (pmol/l)	272 (220, 324)	295 (243, 347)	−23 (−67, 21)	0.298	**<0.001**	0.298
p‐renin (pg/mL)	9.2 (7.2,11.2)	9.3 (7.3, 11.3)	−0.1 (−1.2, 1.0)	0.863	**<0.001**	0.897
p‐ANG II (pg/mL)	5.4 (4.2, 6.6)	5.2 (4.0, 6.4)	0.21 (−0.37, 0.79)	0.476	**<0.001**	0.599
p‐AVP (pg/mL)	0.16 (0.13, 0.18)	0.16 (0.14, 0.18)	0.00 (−0.02, 0.02)	0.979	0.258	0.235
p‐BNP (pmol/l)	4.3 (3.4, 5.3)	5.0 (4.1, 6.0)	−0.66 (−1.35, 0.02)	0.059	0.141	0.995

*Note*: Treatment effects were analyzed using linear mixed‐effects models including fixed effects of treatment, time, and their interaction, with participant ID as a random effect. Models were additionally adjusted for sex and treatment sequence (randomization order). All variables are presented as estimated marginal means (95% CI) and absolute mean differences (potassium nitrate minus placebo) with 95% CI. *p*‐values for treatment, time, and treatment × time interaction were obtained from Type III tests using the Kenward‐Roger approximation. No adjustment for multiple comparisons was applied to these model‐derived overall effects. A two‐sided *p*‐value <0.05 was considered statistically significant. Bold *p*‐values indicate statistical significance (*p* < 0.05).

Abbreviations: ANG II, angiotensin II; AVP, arginine vasopressin; BNP, brain natriuretic peptide; CH_2_O, free water clearance; DBP, diastolic blood pressure; ENaC, epithelial sodium channel; FE_K_, fractional excretion of potassium; FE_Na_, fractional excretion of sodium; GFR, glomerular filtration rate; NCC, sodium‐chloride cotransporter; SBP, systolic blood pressure; UKV, urinary potassium excretion; UNaV, urinary sodium excretion.

### 3.3 Systemic hemodynamic

**FIGURE 2:**
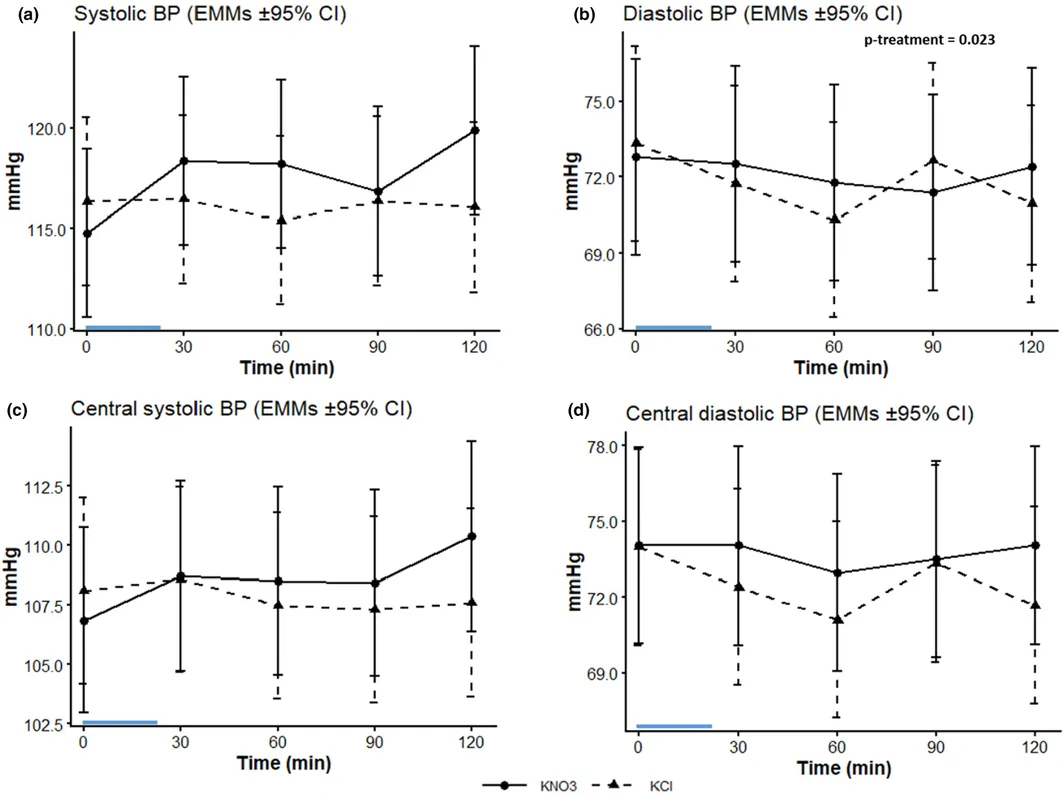
Brachial and central blood pressure responses to acute saline infusion following 4 days of potassium nitrate versus placebo. The 1 L isotonic saline infusion was administered from 0 to 20 min. Time courses are presented as estimated marginal means ± 95% confidence intervals for (a) brachial systolic blood pressure, (b) brachial diastolic blood pressure, (c) central systolic blood pressure, and (d) central diastolic blood pressure, measured every 30 min during the examination (*n* = 18). The shaded bar indicates the saline infusion period. Solid lines represent potassium nitrate and dashed lines represent placebo; vertical bars indicate 95% confidence intervals. Estimated marginal means were derived from linear mixed‐effects models including treatment, time, treatment period, sex, and the treatment × time interaction, with a subject‐specific random intercept. No post hoc comparisons are displayed in the figures.

Figure 2, Table 2 show systemic hemodynamic responses to the saline infusion. Brachial systolic BP tended to be higher during KNO_3_ treatment compared with placebo (*p* = 0.052). While brachial systolic BP did not differ between treatments at baseline or during the early phase after saline infusion, at the 120‐min time point systolic BP was significantly higher during KNO_3_ treatment compared with placebo (mean difference 3.8 mmHg, Sidak‐adjusted *p* = 0.030). After saline central diastolic BP was modestly but significantly higher during KNO_3_ treatment compared with placebo (mean difference 1.2 mmHg, *p* = 0.023). No significant differences were observed for brachial diastolic BP or central systolic BP after saline (*p* = 0.436 and *p* = 0.335, respectively). Heart rate did not differ between KNO_3_ and placebo treatment after saline infusion (*p* = 0.353).

### 3.4 The nitric oxide system

**FIGURE 3:**
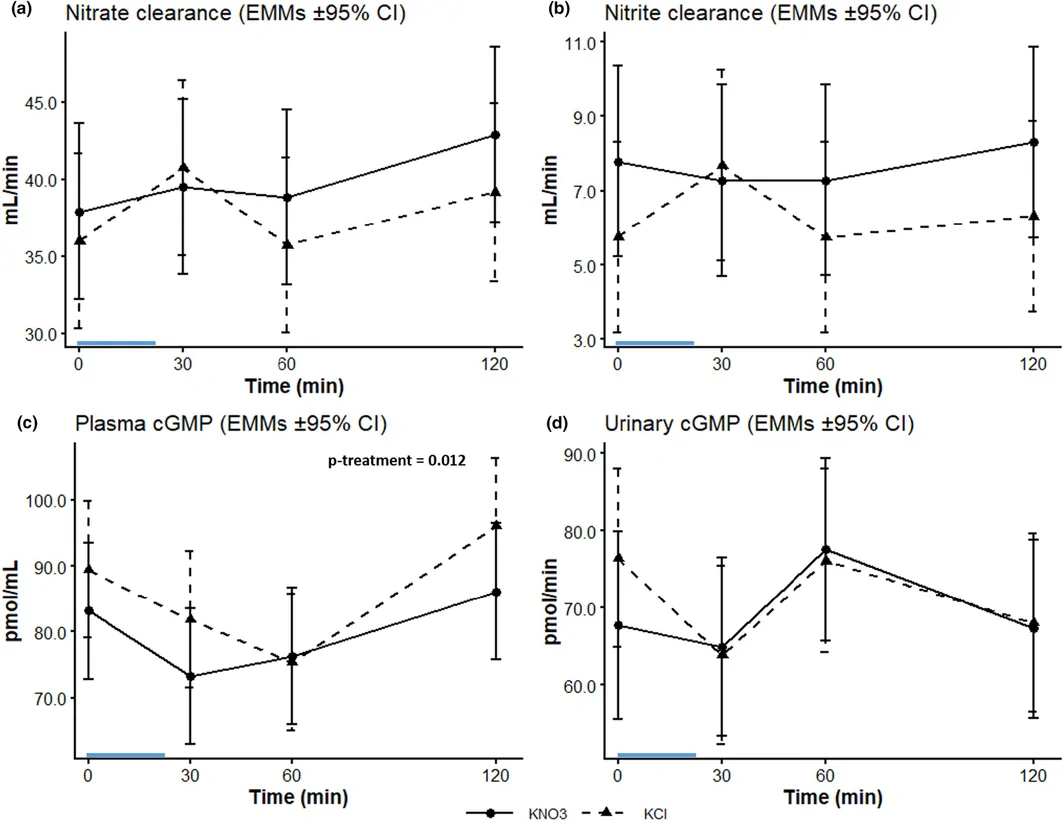
Nitric oxide pathway responses to acute saline infusion following 4 days of potassium nitrate versus placebo. The 1 L isotonic saline infusion was administered from 0 to 20 min. Time courses are presented as estimated marginal means ± 95% confidence intervals for (a) nitrate clearance and (b) nitrite clearance (*n* = 14), (c) plasma cyclic guanosine monophosphate (cGMP) (*n* = 18), and (d) urinary cGMP (*n* = 14). The shaded bar indicates the saline infusion period. Solid lines represent potassium nitrate and dashed lines represent placebo; vertical bars indicate 95% confidence intervals. Estimated marginal means were derived from linear mixed‐effects models including treatment, time, treatment period, sex, and the treatment × time interaction, with a subject‐specific random intercept. No post hoc comparisons are displayed in the figures.

Figure 3, Table 3 summarize responses of the NO pathway. Despite markedly elevated plasma nitrate and nitrite concentrations during KNO_3_ treatment, plasma cGMP concentrations were significantly lower compared with placebo after saline infusion (mean difference −5.9 pmol/mL, *p* = 0.012). Urinary nitrate and nitrite excretion rates were substantially higher following KNO_3_ treatment, reflecting increased substrate availability, whereas nitrate and nitrite clearance did not differ significantly between treatments and after saline (*p* = 0.210 and *p* = 0.101, respectively).

**TABLE 3:** Effects of potassium nitrate versus placebo on nitric oxide metabolites and renal handling during saline loading.

	Potassium nitrate	Placebo	Mean difference (95% CI)	Treatment	Time	Treatment × time
Plasma nitric oxide pathway during saline loading (*n* = 18)						
p‐nitrate (μmol/L)^a^	535 [473, 605]	14 [12, 16]	31 [28, 37]	**<0.001**	**<0.001**	0.304
p‐nitrite (μmol/L)	0.37 (0.32, 0.43)	0.14 (0.08, 0.19)	0.24 (0.20, 0.27)	**<0.001**	0.142	0.165
p‐cGMP (pmol/mL)	80 (71, 89)	86 (77, 95)	−5.9 (−10.5, −1.3)	**0.012**	**<0.001**	0.352
Urinary nitric oxide pathway during saline loading (*n* = 14)						
u‐nitrate (μmol/min)^a^	22 [19, 26]	0.51 [0.43, 0.60]	38 [34,43]	**<0.001**	0.100	0.176
u‐nitrite (nmol/min)	2.44 (2.11, 2.76)	0.81 (0.49, 1.14)	1.62 (1.34, 1.91)	**<0.001**	0.688	0.441
u‐cGMP (pmol/min)	69 (62, 77)	71 (64, 79)	−1.7 (−9.3, 5.8)	0.649	0.112	0.761
Nitrate clearance (mL/min)	40 (35, 44)	38 (33, 42)	1.9 (−1.1, 4.9)	0.210	0.121	0.628
Nitrite clearance (mL/min)	7.7 (5.8, 9.5)	6.4 (4.5, 8.3)	1.3 (−0.26, 2.84)	0.101	0.776	0.621
FE_nitrate_ (%)	43 (38, 49)	39 (33, 44)	4.4 (−2.4, 11.2)	0.206	**0.003**	0.903
FE_nitrite_ (%)	7.8 (5.8, 9.7)	6.1 (4.2, 8.1)	1.6 (−0.48, 3.69)	0.129	0.428	0.993

*Note*: Treatment effects were analyzed using linear mixed‐effects models including fixed effects of treatment, time, and their interaction, with participant ID as a random effect. Models were additionally adjusted for sex and treatment sequence (randomization order). All variables are presented as estimated marginal means (95% CI) and absolute mean differences (potassium nitrate minus placebo) with 95% CI. For variables with skewed distributions*, analyses were performed on log‐transformed data, and results are presented as estimated marginal means (95% CI) with treatment effects expressed as geometric mean ratios (potassium nitrate vs. placebo) with 95% CI. *p* values for treatment, time, and treatment × time interaction were obtained from Type III tests using the Kenward‐Roger approximation. No adjustment for multiple comparisons was applied to these model‐derived overall effects. A two‐sided *p*‐value <0.05 was considered statistically significant. Bold *p*‐values indicate statistical significance (*p* < 0.05).

Abbreviations: p‐, plasma; cGMP, cyclic guanosine monophosphate; FE, fractional excretion; u‐, urinary.

^a^ Variables were log‐transformed prior to analysis to improve model assumptions.

### 3.5 Vasoactive hormones

**FIGURE 4:**
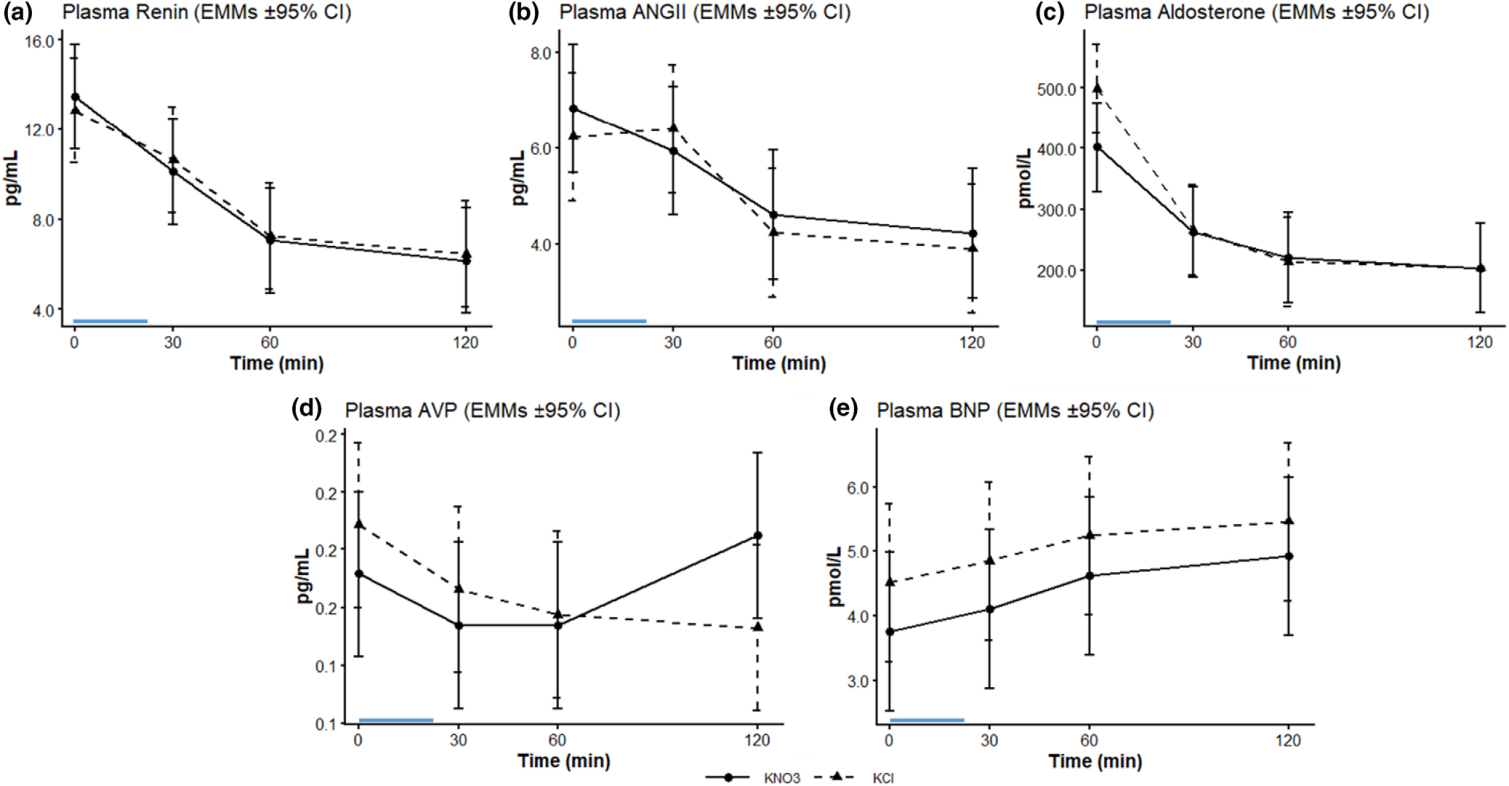
Vasoactive hormonal responses to acute saline infusion following 4 days of potassium nitrate versus placebo. The 1 L isotonic saline infusion was administered from 0 to 20 min. Time courses are presented as estimated marginal means ± 95% confidence intervals for (a) plasma renin, (b) angiotensin II, (c) aldosterone, (d) arginine vasopressin, and (e) brain natriuretic peptide, measured at baseline and at 30, 60, and 120 min (*n* = 18). The shaded bar indicates the saline infusion period. Solid lines represent potassium nitrate and dashed lines represent placebo; vertical bars indicate 95% confidence intervals. Estimated marginal means were derived from linear mixed‐effects models including treatment, time, treatment period, sex, and the treatment × time interaction, with a subject‐specific random intercept. No post hoc comparisons are displayed in the figures.

Figure 4, Table 2 show vasoactive hormonal responses to saline infusion. Plasma renin, angiotensin II, and aldosterone responses decreased during the examination day in response to saline (all *p* < 0.001), with no significant differences between KNO_3_ and placebo treatment (*p* = 0.720, *p* = 0.716, and *p* = 0.230, respectively). Plasma AVP responses did not differ between treatments overall (*p* = 0.979), although a small, isolated difference in response from baseline was observed at the 120‐min time point after saline (Sidak‐adjusted *p* = 0.033). Plasma BNP showed a non‐significant tendency toward lower levels during KNO_3_ treatment compared with placebo (*p* = 0.059).

## 4 DISCUSSION

We were not able to demonstrate an increased GFR or natriuretic response to saline infusion during KNO_3_ as compared to placebo. Further, we found a modest hemodynamic response showing an unexpectedly higher central diastolic BP and tendency toward a higher brachial systolic BP during KNO_3_ as compared to placebo. A response, which could be indicative of diminished endogenous NO production following KNO_3_ treatment, supported by a lower level of cGMP in plasma (Figure 3). Several regulatory mechanisms may contribute to these findings.

Firstly, an acute volume expansion would be expected, through increased endothelial shear stress, to stimulate endogenous NO formation, typically reflected by increased levels of the second messenger cGMP. In the present study, however, plasma cGMP concentrations were significantly lower during KNO_3_ treatment compared with placebo, despite markedly elevated plasma nitrate and nitrite levels. This unexpected pattern of lower plasma cGMP may suggest an attenuation of the endogenous NO production during high nitrate exposure. These observations are consistent with previous experimental and clinical studies demonstrating paradoxical vascular effects of high‐dose nitrate: In rats, low‐dose nitrate lowered BP, whereas high‐dose nitrate increased BP and reduced NOS activity (Carlström et al., 2015). In humans, higher doses have likewise failed to demonstrate further reductions in BP (Babateen et al., 2023). Our results support the concept that exogenous nitrate may dampen the endogenous NO response to acute physiological stressors such as volume expansion. One proposed mechanism is feedback inhibition of endogenous NOS activity by elevated circulating nitrite levels, which inhibits NOS‐derived NO production (Bahadoran et al., 2020; Buga et al., 1993; Lang Jr. et al., 2007). During the KNO_3_ treatment, plasma nitrite concentrations exceeded physiological levels, which may have limited the capacity for further NO generation in response to the acute volume expansion.

Secondly, we examined the involvement of AVP in the hemodynamic response to saline infusion. AVP responses increased modestly but significantly toward the end of the study period during KNO_3_ treatment compared with placebo. This delayed difference in response from baseline may reflect baroreceptor‐mediated activation of counter‐regulatory neurohormonal pathways in response to altered BP. Alternatively, high nitrate exposure itself may directly stimulate AVP release, as previously speculated (Eriksson et al., 1982; Goyer et al., 1994; Lutz‐Bucher & Koch, 1994; Manning Jr. et al., 1994; Ota et al., 1993; Yasin et al., 1993). The late timing of the AVP response may also explain why no clear differences in urine output were observed between treatments. This observation may reflect that AVP‐mediated hemodynamic regulation is recruited later during acute volume expansion, in contrast to the rapid compensatory response mediated by endogenous NO production. However, this finding should be interpreted cautiously, as the difference from baseline was observed at a single time point only.

Finally, we examined the acute hemodynamic response mediated by the renin‐angiotensin‐aldosterone system (RAAS). As expected, saline infusion resulted in marked suppression of the RAAS with no evidence that KNO_3_ modified this response. These findings suggest that RAAS activity is unaffected by KNO_3_ treatment and does not appear to be modulated by NO production during the present experimental conditions.

### 4.1 Strengths and limitations

Strengths of this study include the randomized, double blind, placebo‐controlled crossover design, strict dietary standardization of nitrate, nitrite, and sodium intake, and the comprehensive assessment of both renal and systemic physiology. The steady state GFR measurements and detailed vasoactive analyses provided robust physiological data, which are rare in human nitrate research. Limitations include the relatively small sample size and the exploratory nature of several secondary outcomes, which increase the risk of both type I and type II errors. The study population consisted of healthy young adults, limiting the generalizability of the findings to older individuals or patients with chronic diseases. Furthermore, our results should be interpreted in the context of short‐term KNO_3_ treatment. We observed a tendency toward higher sodium excretion during KNO_3_ treatment compared with placebo. Similarly, there was a trend toward increased urinary ENaCγ excretion. These effects might have reached statistical significance with a longer treatment period prior to the saline infusion.

## 5 CONCLUSION

In conclusion, short‐term inorganic KNO_3_ supplementation compared with placebo did not induce a natriuretic response during acute saline loading. Instead, KNO_3_ treatment was associated with a modest increase in central diastolic BP, lower plasma cGMP levels, and a delayed increase in AVP response. These findings suggest that high‐dose nitrate exposure may attenuate the endogenous NO response to acute volume expansion. Further studies are warranted to elucidate the physiological conditions under which KNO_3_ treatment may exert beneficial effects.

## AUTHOR CONTRIBUTIONS

**A. M. Østergaard:** Conceptualization; data curation; formal analysis; funding acquisition; investigation; methodology; project administration; resources; software; supervision; validation; visualization. **J. B. Rosenbæk:** Conceptualization; investigation; methodology. **J. A. Ejlersen:** Data curation; investigation; methodology; resources; software; validation. **F. H. Mose:** Data curation; investigation; methodology; supervision. **J. N. Bech:** Conceptualization; data curation; formal analysis; funding acquisition; project administration; resources; supervision. **M. H. Vrist:** Conceptualization; data curation; formal analysis; investigation; methodology; supervision; validation; visualization.

## FUNDING INFORMATION

Support was provided solely from institutional and departmental sources.

## CONFLICT OF INTEREST STATEMENT

The authors declare no conflicts of interest.

## DISCLOSURE

AI statement: ChatGPT (OpenAI) was used for English language editing and clarity. All analyses, interpretations, and conclusions were performed by the authors.

## CLINICALTRIALS.GOV IDENTIFIER

NCT04755400, 17/02/2021.

## Data Availability

Primary endpoints and baseline data have previously been reported (Østergaard et al., 2023). The datasets used and analyzed during the current study are available from the corresponding author on reasonable request.

## References

[phy270919-bib-0001] Al Therwani, S. , Mose, F. H. , Jensen, J. M. , Bech, J. N. , & Pedersen, E. B. (2014). Effect of vasopressin antagonism on renal handling of sodium and water and central and brachial blood pressure during inhibition of the nitric oxide system in healthy subjects. BMC Nephrology, 15, 100. 24965902 10.1186/1471-2369-15-100 PMC4079642

[phy270919-bib-0002] Babateen, A. M. , Shannon, O. M. , O'Brien, G. M. , Olgacer, D. , Koehl, C. , Fostier, W. , Mathers, J. C. , & Siervo, M. (2023). Moderate doses of dietary nitrate elicit greater effects on blood pressure and endothelial function than a high dose: A 13‐week pilot study. Nutrition, Metabolism, and Cardiovascular Diseases, 33(6), 1263–1267. 10.1016/j.numecd.2023.02.024 36958967

[phy270919-bib-0003] Bahadoran, Z. , Carlström, M. , Mirmiran, P. , & Ghasemi, A. (2020). Nitric oxide: To be or not to be an endocrine hormone? Acta Physiologica (Oxford, England), 229(1), e13443. 31944587 10.1111/apha.13443

[phy270919-bib-0004] Bech, J. N. , Nielsen, C. B. , & Pedersen, E. B. (1996). Effects of systemic NO synthesis inhibition on RPF, GFR, UNa, and vasoactive hormones in healthy humans. The American Journal of Physiology, 270(5 Pt 2), F845–F851. 8928847 10.1152/ajprenal.1996.270.5.F845

[phy270919-bib-0005] Buga, G. M. , Griscavage, J. M. , Rogers, N. E. , & Ignarro, L. J. (1993). Negative feedback regulation of endothelial cell function by nitric oxide. Circulation Research, 73(5), 808–812. 7691429 10.1161/01.res.73.5.808

[phy270919-bib-0006] Carlström, M. , Liu, M. , Yang, T. , Zollbrecht, C. , Huang, L. , Peleli, M. , Borniquel, S. , Kishikawa, H. , Hezel, M. , Persson, A. E. G. , Weitzberg, E. , & Lundberg, J. O. (2015). Cross‐talk between nitrate‐nitrite‐NO and NO synthase pathways in control of vascular NO homeostasis. Antioxidants & Redox Signaling, 23(4), 295–306. 24224525 10.1089/ars.2013.5481 PMC4523008

[phy270919-bib-0007] Charkoudian, N. , Martin, E. A. , Dinenno, F. A. , Eisenach, J. H. , Dietz, N. M. , & Joyner, M. J. (2004). Influence of increased central venous pressure on baroreflex control of sympathetic activity in humans. American Journal of Physiology. Heart and Circulatory Physiology, 287(4), H1658–H1662. 15191897 10.1152/ajpheart.00265.2004

[phy270919-bib-0008] Convertino, V. A. , Benjamin, B. A. , Keil, L. C. , & Sandler, H. (1984). Role of cardiac volume receptors in the control of ADH release during acute simulated weightlessness in man. The Physiologist, 27(6 Suppl), S51–S52. 11539013

[phy270919-bib-0009] Eriksson, S. , Appelgren, B. , Rundgren, M. , & Andersson, B. (1982). Vasopressin release in response to intracerebroventricular L‐alanine and L‐arginine, and its dependence upon CSF NaCl concentration. Acta Physiologica Scandinavica, 116(1), 75–81. 6818840 10.1111/j.1748-1716.1982.tb10601.x

[phy270919-bib-0010] Frank, H. , Mose, A. E. O.‐K. , & Bech, J. N. (2020). Effect of furosemide on body composition and urinary proteins from tubular sodium and aquaporin channels ‐ a randomized controlled trial. Physiological Reports. Effect of furosemide on body composition and urinary proteins that mediate tubular sodium and sodium transport—A randomized controlled trial, 8. 10.14814/phy2.14653 PMC7757674 33356004

[phy270919-bib-0011] Gabbai, F. B. , & Blantz, R. C. (1999). Role of nitric oxide in renal hemodynamics. Seminars in Nephrology, 19(3), 242–250. 10226330

[phy270919-bib-0012] Goyer, M. , Bui, H. , Chou, L. , Evans, J. , Keil, L. C. , & Reid, I. A. (1994). Effect of inhibition of nitric oxide synthesis on vasopressin secretion in conscious rabbits. The American Journal of Physiology, 266(2 Pt 2), H822–H828. 7511350 10.1152/ajpheart.1994.266.2.H822

[phy270919-bib-0013] Jensen, J. M. , Mose, F. H. , Bech, J. N. , Nielsen, S. , & Pedersen, E. B. (2013). Effect of volume expansion with hypertonic‐ and isotonic saline and isotonic glucose on sodium and water transport in the principal cells in the kidney. BMC Nephrology, 14, 202. 24067081 10.1186/1471-2369-14-202 PMC3849534

[phy270919-bib-0014] Jensen, J. M. , Mose, F. H. , Kulik, A. E. , Bech, J. N. , Fenton, R. A. , & Pedersen, E. B. (2014). Abnormal urinary excretion of NKCC2 and AQP2 in response to hypertonic saline in chronic kidney disease: An intervention study in patients with chronic kidney disease and healthy controls. BMC Nephrology, 15, 101. 24970686 10.1186/1471-2369-15-101 PMC4094915

[phy270919-bib-0015] Kanno, K. , Hirata, Y. , Emori, T. , Ohta, K. , Eguchi, S. , Imai, T. , & Marumo, F. (1992). L‐arginine infusion induces hypotension and diuresis/natriuresis with concomitant increased urinary excretion of nitrite/nitrate and cyclic GMP in humans. Clinical and Experimental Pharmacology & Physiology, 19(9), 619–625. 1327594 10.1111/j.1440-1681.1992.tb00514.x

[phy270919-bib-0016] Kapil, V. , Khambata, R. S. , Robertson, A. , Caulfield, M. J. , & Ahluwalia, A. (2015). Dietary nitrate provides sustained blood pressure lowering in hypertensive patients: A randomized, phase 2, double‐blind, placebo‐controlled study. Hypertension, 65(2), 320–327. 25421976 10.1161/HYPERTENSIONAHA.114.04675 PMC4288952

[phy270919-bib-0017] Kapil, V. , Milsom, A. B. , Okorie, M. , Maleki‐Toyserkani, S. , Akram, F. , & Rehman, F. (2010). Inorganic nitrate supplementation lowers blood pressure in humans: Role for nitrite‐derived NO. Hypertension, 56(2), 274–281. 20585108 10.1161/HYPERTENSIONAHA.110.153536

[phy270919-bib-0018] Kielstein, J. T. , Impraim, B. , Simmel, S. , Bode‐Böger, S. M. , Tsikas, D. , Frölich, J. C. , Hoeper, M. M. , Haller, H. , & Fliser, D. (2004). Cardiovascular effects of systemic nitric oxide synthase inhibition with asymmetrical dimethylarginine in humans. Circulation, 109(2), 172–177. 14662708 10.1161/01.CIR.0000105764.22626.B1

[phy270919-bib-0019] Lang, J. D., Jr. , Teng, X. , Chumley, P. , Crawford, J. H. , Isbell, T. S. , Chacko, B. K. , Liu, Y. , Jhala, N. , Crowe, D. R. , Smith, A. B. , Cross, R. C. , Frenette, L. , Kelley, E. E. , Wilhite, D. W. , Hall, C. R. , Page, G. P. , Fallon, M. B. , Bynon, J. S. , Eckhoff, D. E. , & Patel, R. P. (2007). Inhaled NO accelerates restoration of liver function in adults following orthotopic liver transplantation. The Journal of Clinical Investigation, 117(9), 2583–2591. 17717604 10.1172/JCI31892 PMC1950460

[phy270919-bib-0020] Larsen, T. , Mose, F. H. , Bech, J. N. , & Pedersen, E. B. (2012). Effect of nitric oxide inhibition on blood pressure and renal sodium handling: A dose‐response study in healthy man. Clinical and Experimental Hypertension, 34(8), 567–574. 22559218 10.3109/10641963.2012.681727

[phy270919-bib-0021] Lutz‐Bucher, B. , & Koch, B. (1994). Evidence for an inhibitory effect of nitric oxides on neuropeptide secretion from isolated neural lobe of the rat pituitary gland. Neuroscience Letters, 165(1–2), 48–50. 7517025 10.1016/0304-3940(94)90706-4

[phy270919-bib-0022] Mancia, G. , & Grassi, G. (2014). The autonomic nervous system and hypertension. Circulation Research, 114(11), 1804–1814. 24855203 10.1161/CIRCRESAHA.114.302524

[phy270919-bib-0023] Manning, R. D., Jr. , Hu, L. , & Williamson, T. D. (1994). Mechanisms involved in the cardiovascular‐renal actions of nitric oxide inhibition. Hypertension, 23(6 Pt 2), 951–956. 8206634 10.1161/01.hyp.23.6.951

[phy270919-bib-0024] Mose, F. H. , Jensen, J. M. , Therwani, S. , Mortensen, J. , Hansen, A. B. , Bech, J. N. , & Pedersen, E. B. (2015). Effect of nebivolol on renal nitric oxide availability and tubular function in patients with essential hypertension. British Journal of Clinical Pharmacology, 80(3), 425–435. 25778445 10.1111/bcp.12627 PMC4574828

[phy270919-bib-0025] Mose, F. H. , Jörgensen, A. N. , Vrist, M. H. , Ekelöf, N. P. , Pedersen, E. B. , & Bech, J. N. (2019). Effect of 3% saline and furosemide on biomarkers of kidney injury and renal tubular function and GFR in healthy subjects ‐ a randomized controlled trial. BMC Nephrology, 20(1), 200. 31159750 10.1186/s12882-019-1342-x PMC6545674

[phy270919-bib-0026] Nielsen, C. B. , Eiskjaer, H. , & Pedersen, E. B. (1993). Enhanced renal production of cyclic GMP and reduced free water clearance during sodium nitroprusside infusion in healthy man. European Journal of Clinical Investigation, 23(6), 375–381. 8393795 10.1111/j.1365-2362.1993.tb02039.x

[phy270919-bib-0027] Østergaard, A. M. , Langaa, S. S. , Vrist, M. H. , Mose, F. H. , Bech, J. N. , Fynbo, C. A. , Theil, J. , & Ejlersen, J. A. (2021). Technetium‐99m‐MAG3 and technetium‐99m‐DTPA: Renal clearance measured by the constant infusion technique ‐ old news? Clinical Physiology and Functional Imaging, 41(6), 488–496. 34418886 10.1111/cpf.12724

[phy270919-bib-0028] Østergaard, A. M. , Vrist, M. H. , Rosenbæk, J. B. , Ejlersen, J. A. , Mose, F. H. , & Bech, J. N. (2023). The effect of orally administered nitrate on renal function and blood pressure in a randomized, placebo‐controlled, crossover study in healthy subjects. Nitric Oxide, 134‐135, 1–9. 10.1016/j.niox.2023.03.001 36906115

[phy270919-bib-0029] Ota, M. , Crofton, J. T. , Festavan, G. T. , & Share, L. (1993). Evidence that nitric oxide can act centrally to stimulate vasopressin release. Neuroendocrinology, 57(5), 955–959. 8413832 10.1159/000126459

[phy270919-bib-0030] Pedersen, E. B. , Danielsen, H. , & Spencer, E. S. (1984). Effect of indapamide on renal plasma flow, glomerular filtration rate and arginine vasopressin in plasma in essential hypertension. European Journal of Clinical Pharmacology, 26(5), 543–547. 6468469 10.1007/BF00543482

[phy270919-bib-0031] Pedersen, R. S. , Bentzen, H. , Bech, J. N. , & Pedersen, E. B. (2001). Effect of water deprivation and hypertonic saline infusion on urinary AQP2 excretion in healthy humans. American Journal of Physiology. Renal Physiology, 280(5), F860–F867. 11292629 10.1152/ajprenal.2001.280.5.F860

[phy270919-bib-0032] Potter, L. R. (2011). Guanylyl cyclase structure, function and regulation. Cellular Signalling, 23(12), 1921–1926. 21914472 10.1016/j.cellsig.2011.09.001 PMC4856045

[phy270919-bib-0033] Sriram, K. , Laughlin, J. G. , Rangamani, P. , & Tartakovsky, D. M. (2016). Shear‐induced nitric oxide production by endothelial cells. Biophysical Journal, 111(1), 208–221. 27410748 10.1016/j.bpj.2016.05.034 PMC4944664

[phy270919-bib-0034] Yasin, S. , Costa, A. , Trainer, P. , Windle, R. , Forsling, M. L. , & Grossman, A. (1993). Nitric oxide modulates the release of vasopressin from rat hypothalamic explants. Endocrinology, 133(3), 1466–1469. 7689960 10.1210/endo.133.3.7689960

